# Supplementary material for: TV News and COVID-19: Media Influence on Healthy Behavior in Public Spaces
Source: Int J Environ Res Public Health. 2021 Feb 15;18(4):1879. doi: 10.3390/ijerph18041879 (PMC7919256; doi:10.3390/ijerph18041879)
Supplement: Supplementary file 1 [file ijerph-18-01879-s001.pdf]

Table S1. EFA: Results and factors loadings for "Contagion in the red zones and its consequences".

Item	Factor 1<br>Calming information	Factor 2<br>Alarming information
Infected people have usually only mild symptoms	.828	
Even in red zones most people are fine	.826	
The very high number of people recovered from the infection	.823	
The infection does not produce any symptoms in the majority of people	.806	
Many suspected cases of contagion turned out to be negative	.797	
The very low number of infected people compared to the total population	.742	
Dead people were already sick	.667	
The streets and squares almost empty in red zones		.672
The assault on the supermarkets to make stocks of food		.660
Blocking access to red zones		.656
People walking around in the street with protective masks		.655
The new cases of deaths in Italy		.607
Visits to Italy are not recommended for tourists		.555
The first infected person (patient zero) has not been found yet		. 492
Public transport almost empty in many cities		. 459
The long times for the availability of a vaccine against the virus		. 449
The ease of the spreading of the virus		.438
The number of infected people in Italy		.438
The number of swab-tested people		.421

Eigenvalues	4.70	3.64
Explained variance	24.72	19.18
Mean	3.02	4.35
Cronbach's Alpha	.90	.78

Table S2. EFA: Results and factors loadings of "Preventive measures in green zones".

Item	Factor 1 Individual prevention	Factor 2 Social prevention	Factor 3 Institutional restrictions
The importance of washing your hands often	.843		
The importance of covering your mouth when coughing or sneezing	.842		
The importance of not touching your eyes or mouth with your hands	.813		
The importance of keeping social distance interacting with people	.520		
The numbers to call in case of suspected contagion	.507		
The importance of self-isolation if you think you are infected	.461		
Checking the temperature of people in workplaces		.811	
The sanitization carried out on trains and public transport		.757	
The setting up of tent cities for infected people to be used in case of greater spread of the virus		.661	
The possibility only for close relatives to attend the funeral of a deceased		.657	
Checking the temperature of people at airports		.498	
The closure of museums in some municipalities			.822
The closure of schools and universities in some municipalities			.773
The block of school trips			.757
The blocking of public events (e.g. football matches) in some municipalities			.588
The closure of public exercises in some municipalities at certain time slots			.487
Eigenvalues	4.80	2.20	1.87
Explained variance	30.0	13.7	11.7
Mean	4.98	3.15	4.37
Cronbach's Alpha	.77	.77	.77

Table S3. EFA: Results and factors loadings of "Economic effects of COVID-19".

Item	Factor 1<br>Economic effects	Factor 2<br>Price increase
The estimates on economic decline due to coronavirus	.828	
The difficulties of tourism due to coronavirus	.787	
The overall economic impact of coronavirus	.775	
The negative effect of coronavirus on work activities	.740	
The stock market crash as a result of coronavirus	.617	
The increase in purchases of safe-haven assets among investors on the stock exchange (e.g. gold)	.522	
The rising prices of anti-virus detergents		.937
The rising prices of protective masks		.931
Eigenvalues	3.76	1.36
Explained variance	47.0	17.0
Mean	4.37	4.60
Cronbach's Alpha	.81	.90

Table S4. EFA: Results and factors loadings of "Emotions".

Item	Factor 1<br>Fear	Factor 2<br>Trust in authorities
I'm afraid that coronavirus can spread widely in my city/town	.914	
I'm afraid that coronavirus can spread widely in Italy	.911	
I'm afraid that coronavirus can infect any person I know	.904	
I'm afraid of being infected with coronavirus	.887	
The Italian Regions can effectively face the coronavirus emergency		.897
The Italian government has the competence to control the coronavirus emergency		.823
The Italian municipalities can adopt the right measures against coronavirus emergency		.794
EU has the right means to face the coronavirus emergency		.714
Eigenvalues	3.33	2.59
Explained variance	41.6	32.3
Mean	3.42	3.26
Cronbach's Alpha	.93	.82

Table S5. EFA: Results and factors loadings of "Change in use of public spaces".

Item	Factor 1<br>Change in use of public spaces
I go to cinemas or theaters less often than before	.884
I avoid staying in crowded places outdoors more than before	.850
I go to restaurants or public places less often than before	.845
I use public transport in a different way	.789
I have increased the purchase of groceries	.746
I have increased purchases of protective devices, e.g. face masks, sanitizing gel	.729
I have decided to change my work habits	.710
I have changed my plans for business trips or holidays	.690
Eigenvalues	4.91
Explained variance	61.4
Mean	1.58
Cronbach's Alpha	.91
